# Visual encoding of partial unknown shape boundaries

**DOI:** 10.3934/Neuroscience.2018.2.132

**Published:** 2018-05-16

**Authors:** Hannah Nordberg, Michael J Hautus, Ernest Greene

**Affiliations:** 1 Department of Psychology, University of Southern California, Los Angeles, California USA; 2 The School of Psychology, University of Auckland, Auckland New Zealand, California USA

**Keywords:** shape recognition, shape encoding, boundary marking

## Abstract

Prior research has found that known shapes and letters can be recognized from a sparse sampling of dots that mark locations on their boundaries. Further, unknown shapes that are displayed only once can be identified by a matching protocol, and here also, above-chance performance requires very few boundary markers. The present work examines whether partial boundaries can be identified under similar low-information conditions. Several experiments were conducted that used a match-recognition task, with initial display of a target shape followed quickly by a comparison shape. The comparison shape was either derived from the target shape or was based on a different shape, and the respondent was asked for a matching judgment, i.e., did it “match” the target shape. Stimulus treatments included establishing how density affected the probability of a correct decision, followed by assessment of how much positioning of boundary dots affected this probability. Results indicate that correct judgments were possible when partial boundaries were displayed with a sparse sampling of dots. We argue for a process that quickly registers the locations of boundary markers and distills that information into a shape summary that can be used to identify the shape even when only a portion of the boundary is represented.

## 1. Introduction

Completion can and does occur only if the “seen” part implies a whole of which it is a part.

Wilhelm Fuchs, 1921 [1].

Our goal is to better understand how the visual system is able to encode the shapes of objects, this being a first step toward classification, comparison, and recognition. A viable theory should be based on concepts that are biologically plausible and should provide for the range of judgments of which humans are capable. The processing skills should include the ability to identify shapes irrespective of where they are placed within the visual field, or if the viewing distance has altered the size of the retinal image. There should be at least some ability to compensate for rotation of the shape.

Computer scientists and engineers have developed numerous concepts for how to effectively encode shapes, and can often demonstrate the utility of their methods by accomplishing real-world tasks. One concept that has become popular in recent years has been to use metric information, specified in relation to a centroid, for object and shape identification. A sampling of applications using centroid-based encoding could include: Measuring attributes of 2D and 3D curves [2]; characterizing silhouette-like shapes [3],[4]; personal identification on the basis of hand shape [5]; recognition of 3D objects in cluttered scenes [6]; object tracking [7]; head and face recognition under “wild” conditions [8]; reading of traffic signs for driver assist with translation, rotation, and size invariance [9].

It seems clear that centroid-based shape summaries are both effective and robust, but are they biologically plausible? Greene [10]–[12] has suggested that the anatomy and physiology of the retina could provide for centroid-based shape summaries. A key concept is that stimulus-marked locations on the outer boundary of an object could provide angle and distance information through waves of activation that spread from each location, converging on the centroid. The time of arrival of successive waves would be registered at the centroid, such that the resulting moment-by-moment fluctuations of the converging activity would serve as a summary of the shape.

It would be helpful to have a test of the centroid hypothesis; that was one goal of the present effort. The experiments reported here made use of unknown shapes in a match-recognition task that was initially developed by Greene and Hautus [13]. Their protocol displays shapes as a sequence of dots that are briefly flashed on an LED array. The first shape to be shown on a given trial is the target shape, which provides a continuous string of boundary dots. The comparison shape that follows is either a low-density (sparse) sequence of dots that matches the target shape, or is a non-matching shape. Low-density shapes are used so that participant performance will not be perfect, i.e., to avoid a ceiling effect. The respondent sees a given shape only once, either as a target or comparison shape, this being to preclude any role for long-term memory and to keep the focus on the encoding process. The judgments about whether comparison shapes match the targets are analyzed using the methods of signal-detection theory, also known as detection theory. This provides an index of the probability that the decisions are valid with correction for bias.

Whereas Greene and Hautus [13] displayed boundary markers around the full perimeter of each comparison shape, here we are providing markers around only portions of the boundary. This modifies the location of the centroid in relation to the markers, which should impair the ability to see the boundary as a match to the target shape. The disruption should be worse when all the markers are on the same side of the shape and less if markers are symmetrically placed across the midline. The sequence of four experiments was designed to evaluate the ability of respondents to perform the matching judgment where density of markers, amount of marked boundary, and the balance of markers were manipulated as treatment variables.

## 2. Method

### 2.1. Authorization of research and informed consent

Research protocols were approved by the USC Institutional Review Board. A total of 32 respondents were recruited from the USC Psychology Subject pool, eight different respondents providing data in each of the four experiments. Each was informed that participation was voluntary and that they could discontinue at any time and for any reason. All who volunteered to participate completed the experiment for which he or she was recruited.

### 2.2. Display equipment and experimental protocols

Shape stimuli were displayed on a 64 × 64 array of light-emitting diodes (LEDs), designated hereafter as the “display board”. The shape stimuli (described more completely below) were displayed as 10 microsecond (µs) simultaneous flashes of all the dots comprising the shape, or with a sampling of those dots. Viewing distance was 3.5 m, so the visual angle subtended by each dot was 4.92 arc′, dot-to-dot spacing was 9.23 arc′, and total span of the array (center-to-center of outside dots) was 9.80 arc′.

Intensities of the fixation point and of flashes were specified in radiometric units, specifically microwatts per solid angle (μW/sr). The LEDs emitted at a peak wavelength of 630 nm (red). Given the narrow range of wavelengths of LED emissions, likely only L-opsin cones were stimulated.

Experimental protocols were administered by a Mac G4 Cube, programmed with Tk/tcl instructions. These instructions were further interpreted as machine language through a Propox MMnet101 microcontroller with a running speed of 16 Mhz. This system provided flash durations and timing of stimulus displays with a temporal resolution of 1 μs.

### 2.3. Shape inventory and selection of shapes for display

Each experiment drew from an inventory of 480 unknown shapes. This inventory was a bit larger than required by present experiments, but the number allows for a greater number of treatment combinations should they be needed. Each shape was formed as a continuous string of dots that formed a single unbroken loop, like an outline drawing. The dot sequences were constructed with arbitrary turns, arcs, and straight sections, yielding shapes that bear little resemblance to known objects.

The number of boundary dots in the inventory of shapes ranged from 100 to 269, with a mean dot-count of 166. At the 3.5 m viewing distance, this corresponds to a range of 2.0 to 3.5 arc°, with mean distance being 2.6 arc°.

For a given respondent, shapes were chosen at random from the inventory to be used as targets (described more completely below) or for development of stimuli to be compared for matching to these targets. A shape that was chosen for display of all boundary dots or a portion of the boundary dots is generically designated as a “source shape”. Each of the four experiments chose 300 source shapes from the inventory to be used as “target shapes”, and another 150 source shapes to be used for creating non-matching shapes (see below).

### 2.4. Common task conditions

**Figure 1.**
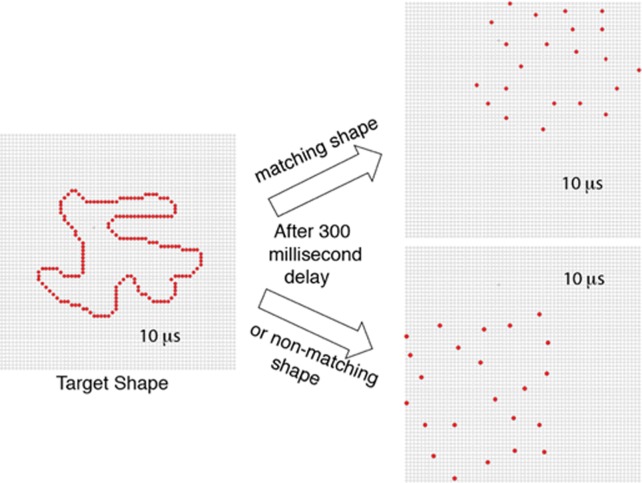
Outline of match-to-sample protocol. In each experiment, a given trial began with display of a target shape that had been randomly selected from the shape inventory. Each dot in the boundary was simultaneously displayed as a 10 µs flash. A comparison shape was displayed 300 ms later, consisting either of a low-density sample of dots from the target shape (designated as matching) or a low-density sample of dots from a different shape (designated as non-matching). The comparison shape was also displayed as a simultaneous 10 µs flash of all sampled dots. Target shapes were centered on the display board, and a given target shape was shown only once to a given respondent. Comparison shapes were displayed at locations that were eccentric to the center.

A match-recognition protocol was used for each of the four experiments; see Figure 1. On a given trial the respondent was presented with two successive displays. First a target shape was shown with simultaneous ultra-brief flashes of all the dots comprising the boundary, i.e., at 100% density. It was positioned with the centroid of the shape being at the center of the display board. In each of the experiments a given target shape was shown only once to a given respondent.

Shortly after display of the target shape, a comparison shape was shown. A comparison shape consisted of either a low-density sampling of dots from the target shape, designated as “matching”, or a low-density sampling of dots from a different source shape, designated as “non-matching”. In addition to being low in dot density, comparison shapes (matching or non-matching) were also subject to selective elimination of boundary sectors to evaluate judgments of shape matching where only a portion of the source-shape area was enclosed.

For each of the experiments, each trial was preceded by display of a fixation point that consisted of steady emission from four dots that were at the center of the display board. Emission intensity was 0.2 μW/sr. The fixation dots were extinguished 200 ms prior to display of the target shape. Dots of the target shape were simultaneously flashed for 10 μs at an intensity of 1000 μW/sr. A matching or non-matching comparison shape was displayed 300 ms later at the same intensity and for the same duration. Ambient room illumination was 10 lx, so the flashed dots were very conspicuous.

As indicated above, target shapes were displayed at the center of the board. Comparison shapes were displaced from that center at randomly determined eccentric locations. To this end, the centroid of the source shape was placed at a distance of 20 dots (3 arc°) and at a randomly chosen angle. Then the boundary dots were moved horizontally or vertically as needed to reposition the most extreme boundary dots to the edges of the LED array.

Each trial was initiated by the experimenter by clicking an on-screen button, eliciting display of the target and comparison shapes. This was not a speeded task, but respondents generally judged the display and voiced a response—same or different—within a second or two. This answer was recorded by the experimenter by clicking a corresponding on-screen button. The computer recorded what had been displayed as well as the response. The experimenter was given no information about which shapes or conditions had been displayed or whether the response was correct, nor did the respondent receive any information about the validity of the answer.

### 2.5. Individual experimental protocols

**Figure 2.**
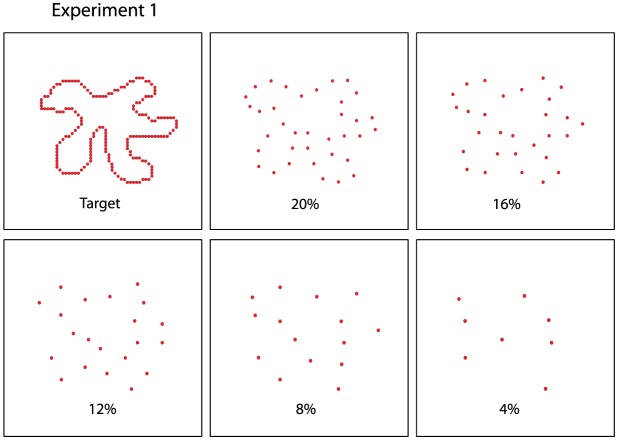
Density levels of the comparison shapes displayed in Experiment 1. If the shape in the left panel was chosen as a target, one of the density levels in the other five panels could be chosen to provide a matching comparison shape, or a non-matching shape might be displayed at one of the five density levels. The dots used to illustrate low-density boundary markers have been resized up to adjust for saliency in the illustration. The actual LED-flashed dots, seen in a dim room, were very readily perceived.

Experiment 1 provided basic assessment of how dot density affects matching judgments under the present display conditions. Density of comparison stimuli was varied across five levels, specifically 4, 8, 12, 16, and 20%, as illustrated in Figure 2. The dots that were displayed from a given source shape were specified by first randomly choosing which boundary dot to use as a starting point, then moving through successive boundary locations selecting which dots to include for display at the specified density. An algorithm was used that maximized spacing of the sample, as prior research had found that uniform spacing enhances recognition of shapes [21]. For each of the five density treatment levels, a given respondent judged 30 matching trials and 30 non-matching trials, for a total of 300 trials.

**Figure 3.**
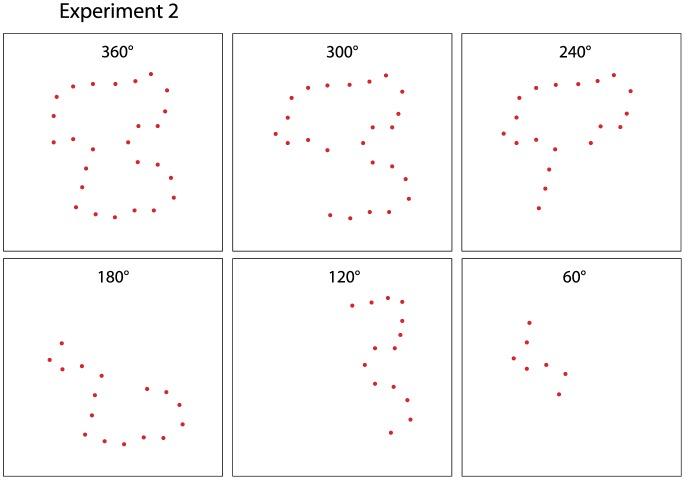
Five sector sizes that pivoted on the centroid of the target shape in Experiment 2. The angle that bisected the sector was chosen at random. The size and positioning of the sector determined whether a low-density boundary dot would be displayed. Boundary dots lying within the inclusive zone were sampled to provide a 16% density.

All comparison stimuli in Experiment 2 were displayed at a density of 16%. The amount of boundary perimeter to display was varied in 60° increments, as follows. Beginning with source shape, the centroid was established and angles were positioned every 60° to partition the shape into sectors. Treatment levels provided for display of dots within one to six sectors, i.e., with sector sizes being 60, 120, 180, 240, 300, and 360° (see Figure 3). Only boundary dots fell within the chosen sector were displayed, with deletion of any isolated sections of boundary even those dots fell within the sector that was specified. With six levels of sector size, there were 25 matching stimuli and 25 non-matching stimuli at each level, providing 300 total trials for a given respondent. As in Experiment 1, the comparison stimulus on a given trial was positioned eccentric to the center of the board, with the centroid of the original source shape being place at a 20-dot distance from the center of the board and at a random angle.

**Figure 4.**
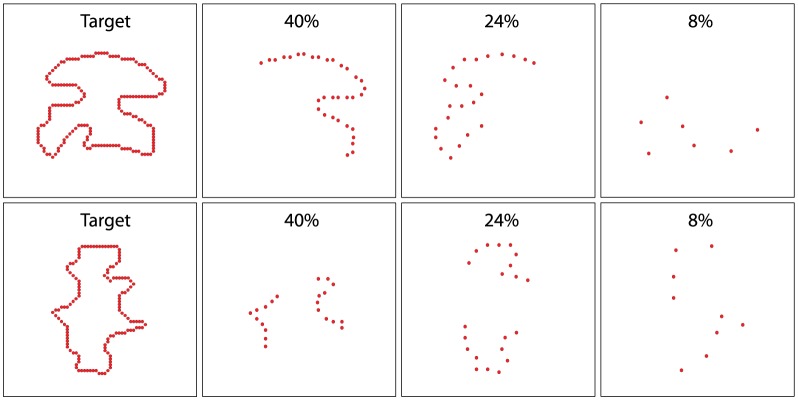
Matching comparison shape density levels in Experiment 3 and 4. Experiment 3 varied the bisecting angle of a 180° sector at random, and provided for five density levels within the sector that might be chosen for display. The upper panels illustrate three of the five levels that might be shown as a matching comparison shape. Experiment 4 provided for two 90° sectors that were centered on the bisecting axis. The same five density levels were tested, with the lower panels illustrating three of the levels that might be chosen for a matching comparison shape.

Experiment 3 displayed partial boundaries as comparison stimuli using a single sector size of 180°. Whereas Experiment 2 specified the sectors as fixed angular departures from 0°, here the sectors were specified relative to an angle that varied. For a given trial, an angle was randomly chosen, and the boundary dots to be displayed fell within the 90° sector on each side of that angle. Here also, any isolated boundary sections were deleted. Therefore, each comparison stimulus provided a string of boundary markers that spanned 180°, but with the choice of which portion of the source-shape boundary to use was determined at random (see Figure 4). Dot density was varied, using density levels that were double the percentages of Experiment 1, namely: 8, 16, 24, 32, and 40%. A given respondent saw thirty matching and thirty non-matching comparison shapes at each of the five density levels for a total of 300 trials. Here also, the centroid of the source shape was used to determine the eccentric location at which the comparison stimuli would be placed.

Experiment 4 was identical in design to Experiment 3 except the boundary dots to be displayed were positioned in two opposing 90° sectors. For a given comparison stimulus, after choosing the random angle that would bisect the sector, the sector size was set at 90° (45° on each side of the angle) and this zone was mirrored on the opposite side of the centroid. This provided a total amount of boundary that was comparable to a 180° sector. The difference was that Experiment 4 provided boundary markers on both sides of the centroid, whereas the boundary markers of Experiment 3 were all on the same side (see Figure 4). The significance of this difference will be discussed subsequently.

### 2.6. Respondent judgments

Respondents were instructed not to move their gaze from the fixation point. However, it is unlikely that changes of gaze would modify response performance, because each display trial was completed in 300 ms and the respondent had no information about where the comparison stimulus would appear. Respondents were told that the first display would show the outline of a shape using a string of dots, and the second display would show a low-density version of the target boundary or a low-density boundary of a different shape. Instructions for the last three experiments included clarification that the stimulus might provide only a low-density partial boundary. Respondents were told that they should say “same” if the comparison stimulus displayed dots that could have come from the target shape, and should say “different” if the stimulus likely came from a different shape. Respondents appeared to understand the instructions and task performance on all four experiments is consistent with proper interpretation of task demands.

### 2.7. Quantitative analysis of data

Once task protocols were established through pilot work, each respondent who volunteered for testing was able to complete his or her test session without difficulty, and all data that was collected for a given experiment has been included in the quantitative analysis. The analysis of data was based on detection theory, as detailed in an earlier report [13].

For all experiments, each respondent provided judgments at each level of the independent variable yielding false-alarm (F) and hit (H) rates. Each rate was based on 30 trials for Experiments 1–3 and on 25 trials for Experiment 4. In each case, an estimate of d′ was determined from each (F, H) pair together with its associated variance. Results are reported here at the group level rather than for individuals. To obtain these we averaged individual d′ estimates across respondents for each level of the independent variable [14]–[20]. The variance associated with these averaged estimates were also calculated and used to generate 95% confidence intervals around the group estimate of d′. These performance measures, including confidence intervals, were then transformed to values of *p*(*c*)_max_. For the index, *p(c)*_max_, a score of 0.50 represents chance performance and a score of 1 indicates judgments that were always correct.

## 3. Results

**Figure 5.**
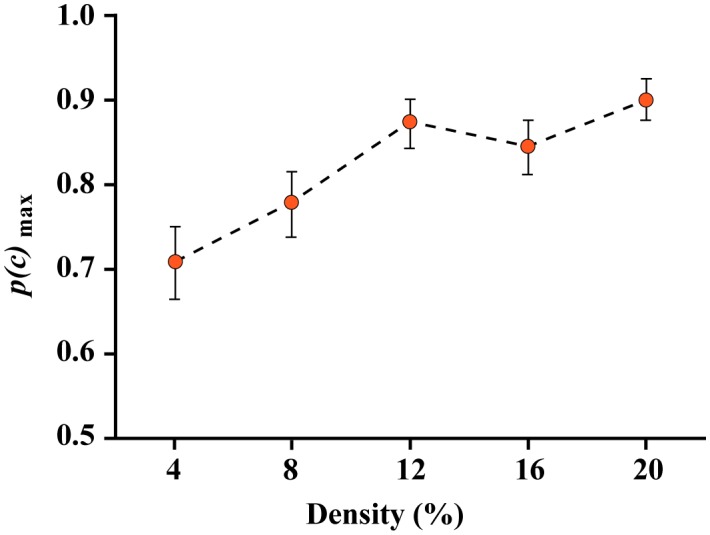
Experiment 1 *p(c)*_max_ values. The *p(c)*_max_ values affirm that subjects provided above-chance decisions about whether the comparison shapes matched the target shapes. The *p(c)*_max_ value provides an unbiased index of correct judgments by using detection theory to adjust the match probability for the number of trials wherein the non-matching shapes were incorrectly judged. Chance performance is at 0.5, so clearly the respondents provided accurate decisions on most trials, even when the comparison shape displayed only 4% of the dots that were provided by the target shape.

For Experiment 1, the mean *p(c)*_max_ values across respondents are plotted in Figure 5, along with 95% confidence intervals. Experiment 1 provided a strong confirmation of prior results that used a very similar experimental protocol [13]. It affirms that an unknown shape consisting only of boundary markers that is seen only once, can be identified when only a small number of the markers are used to elicit recognition. Additionally, the experiment provided an anchor for the subsequent experiments by showing a consistent decrease in the probability of correct decisions as the density of boundary was reduced, i.e., which increased the sparseness of the resulting dot pattern. With a 20% density the unbiased proportion of decisions that were correct was above 0.9. This level of performance decreased as density was reduced, such that at a 4% density of comparison shapes provided *p(c)*_max_ = 0.708. Given that the error bars in Figure 5 for the 4% and 20% conditions do not overlap, this difference is significant.

**Figure 6.**
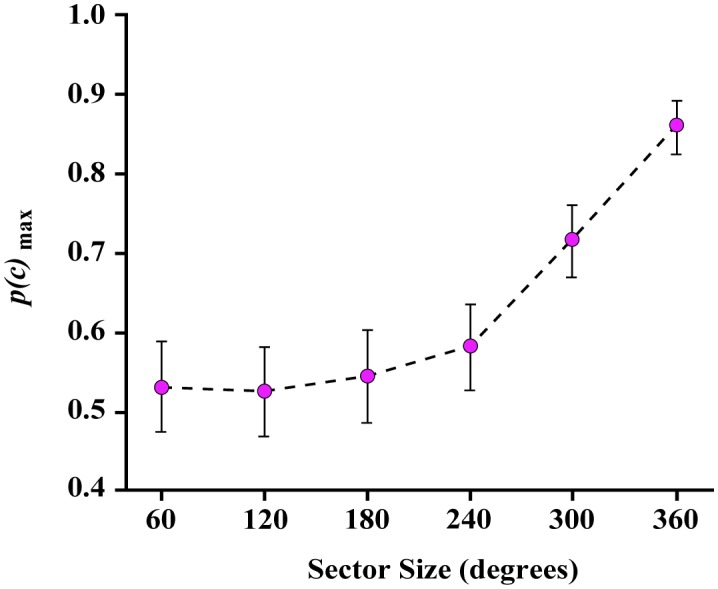
Experiment 2 *p(c)*_max_ values. Experiment 2 varied the amount of boundary that was displayed for target shapes, this being specified as an angular sector size that pivoted on the centroid of each shape. A sector size of 360° provided boundary markers around the full perimeter of the shape. The other panels provide examples of smaller sector angles. Sector size was varied across the six levels, i.e. 60° through 360°, as illustrated, but the exact zone to be displayed was varied at random on each trial. Note that with 16% density the mean judgments were above chance for all sector sizes. The probability of correct judgment began to rise substantially once sector size exceeded 180°.

Experiment 2 tested identification of 180° partial boundaries with the density of comparison shapes set at 16%. Group data are illustrated in Figure 6. The value of *p*(*c*)_max_ was above chance at all tested partial-boundary angles. However, the confidence limits still show lower bounds that were below chance at the first three angles (60°, 120°, and 180°). When a larger portion of the boundary was provided, judgments were significantly above chance. This provides a caution that the encoding and recognition of unknown shapes can be less than reliable if only half of the shape boundary is provided.

**Figure 7.**
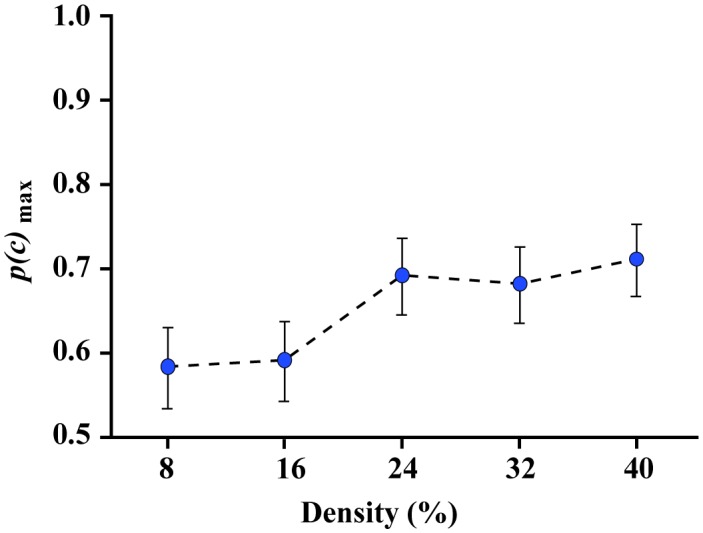
Experiment 3 *p(c)*_max_ values. Experiment 3 used random 180° sectors and varied the density of the boundary markers within a given sector. Density was varied from 8% to 40% and correct judgments were above chance at each of these densities.

Experiment 3 was designed to test how various levels of density would provide for valid shape discrimination of partial boundaries. Each comparison shape was displayed with a partial boundary that was half of the full perimeter, i.e., 180°, and given the findings of Experiment 2, the experiment used a range of densities that was double that provided by Experiment 1, specifically: 8, 16, 24, 32, and 40 percent. Group data are illustrated in Figure 7. Performance was above chance at all levels of density, ranging from just below 60% to just above 70%.

**Figure 8.**
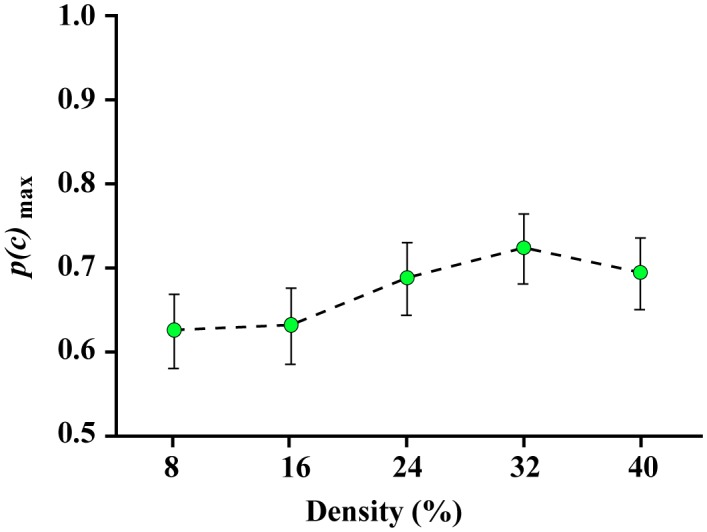
Experiment 4 *p(c)*_max_ values. Experiment 4 used two 90° sectors that were on opposite sides of the centroid. The *p(c)*_max_ values were much the same as found in Experiment 3, which suggests that the ability to make correct judgments depends more on the number of boundary markers displayed and not the degree of symmetry of those markers around the centroid. This deals a blow to encoding concepts that focus on distances and angles relative to the centroid. Alternative concepts are needed to explain how the visual system encodes partial boundaries.

Experiment 4 addressed the question of whether effective shape encoding requires a balance of boundary information on each side of a centroid. Whereas Experiment 3 provided boundary markers from half of the original shape, all being on the same side of the shape, those from Experiment 4 displayed markers that were positioned on opposite sides of the shape. Therefore, the absolute quantity of shape perimeter was the same for both experiments, with those of Experiment 4 being balanced across the centroid of the shape. As can be seen from the group data illustrated in Figure 8, the plot of *p(c)*_max_ across density was very similar to that seen in Experiment 3, so there is no evidence that having a balance of boundary markers provides an advantage for match recognition.

## 4. Discussion

Our major goal is to better understand how shape information is encoded for purposes of recognition. There are several aspects of the current research protocol that contribute to that effort. The use of discrete dots to represent the shape boundary constrains the encoding concepts to those that are biologically plausible. By using ultra-brief flashes, one assures that the shape cues are being delivered to only a single location on the retina. One can ignore ambiguity about whether eye scans or even eye tremors are contributing to the shape-encoding process. Using unknown shapes puts the focus on encoding mechanisms and working memory. Asking for judgment of partial boundaries also limits the concepts that are plausible.

The results show that matching judgments were above chance even when the comparison stimulus provided a low-density sampling of boundary dots. This is consistent with earlier work that questioned whether lines and edges serve as elementary shape cues, and whether activation of orientation-selective neurons in primary visual cortex provide an essential first step for shape encoding. Greene [21] found that common shapes could be identified from a small sampling of boundary dots, and recognition was possible even when the spacing between adjacent dots was greater than the largest receptive fields of orientation selective neurons [22]. Further, boundary dots can be displayed as successive 4-dot subsets, which provide partial cues that can be integrated over time to accomplish shape recognition. Subsets that have sufficient proximity to activate orientation-selective neurons are no more effective as shape cues than are subsets whose members are randomly chosen [23]. Each of the prior reports [21]–[23] provide evidence against the common assumption that lines and edges are elemental shape cues. Further, based on recognition results wherein each dot in the boundary was delivered one-at-a-time, Greene and Ogden [24] inferred that each dot contributes semi-autonomous shape information that can be summed to elicit recognition.

The work cited above studied recognition of known objects. The target shapes used for the current work were unknown. They were displayed only once for a given respondent, and a matching judgment was made quickly after each was shown. This required immediate encoding of shape information and the ability to judge (recognize) matching of shapes based on information stored in working memory.

Greene and Hautus [13] used a similar protocol and found that respondents made above-chance match recognition with comparison shapes providing dot densities as low as 5%. Our first experiment also varied density of the comparison shapes and found that matching judgments were well above chance when they were displayed at 4% density. This supports the challenge to the idea that lines and edges provide elemental shape cues, and reinforces the concept that shapes can be specified using discrete location markers. Further, the earlier study found that decisions were significantly above chance even when comparison shapes were shifted to a different location, magnified in size, or rotated [13]. Matching decisions were rendered quickly after display of the target and comparison shapes, so shape encoding and match-recognition was immediate. This is a challenge to neural connectionist modeling where the encoding process requires many hundreds or thousands of training trials to achieve shape recognition, translation invariance, size invariance, and rotation invariance [25]–[31].

Three of the present experiments asked whether respondents could render above-chance judgments when the comparison shapes provided only a portion of the targets' full boundary. Asking for identification of partial boundaries provides a special challenge to models for how shapes are encoded. Any theory that characterizes shapes using area or volume filling could not readily explain how a partial boundary would be encoded. At best a partial boundary would provide incomplete containment of a given area. An area is even less enclosed where the stimulus is a low-density set of spaced boundary markers.

Greene [10]–[12] proposed that distances among boundary markers, or perhaps from markers to a centroid, could be measured by spreading waves within the retina or tectum. A summary of distances could provide for immediate encoding of marker locations, and depending on other assumptions, that summary could be location, size, and rotation invariant.

The present results cast doubt on the concept that the visual system is registering distances from boundary markers to the centroid. The positioning of boundary markers in the target shape determines where its centroid lies. Display of a matching comparison shape at a different location would provide a similar centroid summary if dot-markers were provided around the full perimeter of the shape. A reduction in dot density might move the centroid by a small amount, but the location would still have substantial correspondence to that provided by the target. However, the centroid for a partial boundary lies at a different position in relation to the markers, and the distance information can differ greatly.

The fundamental problem is especially illustrated by comparing results from Experiments 3 and 4. Experiment 4 used boundary markers that were on opposite sides of the shape. This put the centroid for a given matching partial boundary approximately where it was for the corresponding target shape. However, all the markers for Experiment 3 are on one side of the shape, which changes the location of the centroid for the sampled dots. Matching judgments should have been far worse for Experiment 3 than for Experiment 4. The fact that probabilities of correct decision were similar for the two experiments is at odds with the centroid-encoding hypothesis.

However, the idea that traveling waves within retina and/or tectum can derive shape-encoding information may still be viable. The system might generate waves that pass across the pattern of markers, generating spike activity that specifies how many markers were encountered at successive moments. This approach corresponds somewhat to the latency encoding concept advanced by Hopfield [32]. Hopfield described a way to encode stimulus patterns that have differentials of intensity, e.g., odor components. An oscillation source could bring a more intense element to threshold a bit sooner than one that was less intense, and thus the time-to-fire differences in the resulting population response could be used to specify the intensity profile of the pattern.

Subsequent research by Thorpe and VanRullen also suggested a population coding mechanism for shape recognition. Thorpe and associates [33] had respondents complete a go/no-go task classifying images as containing an animal or not. Analysis of event-related potentials indicated that participants processed the visual stimuli in less than 150 ms. VanRullen and Thorpe [34]–[36] conducted experiments using images in different target categories and found similar results. Regardless of image type, processing speed was incredibly rapid. VanRullen and Thorpe argue that the rapid speed at which image content is registered by the brain provides evidence that the retina only has enough time to generate one or a few spikes, which supports an encoding process such as that proposed by Hopfield [32].

Although Thorpe and VanRullen's work focused on complete, identifiable shapes rather than partial, unknown shapes, the findings have implications for a model of partial boundary encoding. As adapted, a complement of spreading waves, likely from specified directions, could cross the dot pattern and generate differential amplitudes of population spikes as markers were encountered. The shape summary would consist of two or more complex waveforms, each reflecting the density of spikes being elicited from the neuron population. The waveforms from specified travel directions would constitute the shape summary that would be stored for a time in working memory, available for evaluation of the summary from the comparison shape. Recognition would require moment-by-moment matching of waveform amplitudes, which could be thought of as analogous to a sum-of-squared-deviations calculation that is commonly used for comparing two distributions.

This laboratory has recently conducted a computational implementation of the scan-encoding concept [37]. Polling scans were passed across the inventory of unknown shapes to derive histogram summaries, and these summaries were compared to derive a scale of shape similarity. The scale values proved to be significantly able to predict human judgments of similarity of selected pairs, as evidenced by the frequency at which they were judged as being the “same” in the match-recognition task [37]. This supports the concept that polling scans provide a biologically plausible method for encoding shapes.

## 5. Conclusions

The present work provides additional evidence that shape information is quickly encoded and stored in working memory. This allows for immediate comparison of shapes that have not been seen previously. The ability to provide immediate above-chance match recognition of an unknown shape is at odds with neural-network (connectionist) models that require numerous training trials to change connectivity or strength of connection among neuron populations as the basis for encoding and recognition. Our findings also suggest that the encoding of shape information is not based on metric information specified relative to the shape's centroid.

Finding that matching judgments are above chance with low-density partial boundaries calls for an innovative encoding principle. Here we suggest an adaptation of Hopfield's proposal [32] that stimulus information is provided by differentials in spike latency within a stimulus-encoding population of neurons.
